# Rapunzel Syndrome as a Complication of Childhood‐Onset Trichotillomania

**DOI:** 10.1155/crps/6542586

**Published:** 2026-10-01

**Authors:** Ruchika Darapaneni, Zachary Nobile, Regina Reed, Eduardo Espiridion, John Gurski

**Affiliations:** ^1^ Psychiatry Reading Hospital—Tower Health West Reading Pennsylvaina USA towerhealth.org; ^2^ Internal Medicine Reading Hospital—Tower Health West Reading Pennsylvaina USA towerhealth.org; ^3^ Psychiatry Drexel University College of Medicine Philadelphia Pennsylvaina USA drexel.edu

**Keywords:** case report, Rapunzel syndrome, trichophagia, trichotillomania

## Abstract

**Introduction:**

Rapunzel syndrome is a rare and potentially life‐threatening complication of trichotillomania with trichophagia, characterized by the extension of a gastric trichobezoar into the small intestine. Diagnosis is frequently delayed due to nonspecific presenting symptoms, and management requires both surgical intervention and psychiatric treatment to prevent recurrence.

**Patient Case:**

We report the case of a 22‐year‐old woman with childhood‐onset trichotillomania and trichophagia who presented with nonspecific abdominal complaints. Imaging revealed a large intragastric mass, and endoscopy confirmed a dense trichobezoar extending through the pylorus into the duodenum, consistent with Rapunzel syndrome. Endoscopic removal was unsuccessful, and the patient underwent open gastrotomy with intact removal of the mass without complication. Psychiatric evaluation revealed a long history of hairpulling and ingestion behaviors beginning in early childhood, with limited prior psychiatric treatment.

**Discussion:**

This case illustrates the cumulative medical risk of untreated chronic, childhood‐onset trichotillomania with trichophagia, particularly when hair‐ingestion behaviors persist without sustained trichotillomania‐specific behavioral treatment. Its contribution to the literature lies in the detailed longitudinal psychiatric characterization of symptom progression and the consideration of comorbid attention‐deficit/hyperactivity disorder (ADHD), which may be relevant to the hairpulling and ingestion behaviors themselves through attentional dysregulation, impulsivity, impaired inhibitory control, and automaticity.

**Conclusion:**

Rapunzel syndrome represents a severe consequence of chronic hairpulling and ingestion behaviors. Early identification of trichophagia, psychiatric referral, and sustained behavioral treatment are essential to prevent progression to life‐threatening complications and to reduce recurrence following surgical intervention.

## 1. Introduction

Trichotillomania, also known as hairpulling disorder, is a psychiatric condition characterized by recurrent hair‐pulling, resulting in noticeable hair loss and clinically significant distress or functional impairment. The disorder commonly follows a chronic course with waxing and waning symptoms. Trichotillomania frequently co‐occurs with other body‐focused repetitive behaviors such as skin picking and nail biting, and ingestion of pulled hair (trichophagia) may occur in some patients, leading to medical complications including trichobezoar formation [1].

A trichobezoar is a mass of ingested hair that accumulates in the stomach and may extend into the small intestine. When this extension progresses beyond the pylorus into the bowel, the condition is termed “Rapunzel Syndrome,” an extremely rare but well‐described complication of trichophagia in the published medical literature.

Rapunzel syndrome predominantly affects adolescents and young females, reflecting the epidemiologic distribution of trichotillomania and trichophagia. Patients typically present with nonspecific gastrointestinal symptoms, including abdominal pain, nausea, or vomiting, which may delay diagnosis until serious complications develop. Reported complications include bowel obstruction, gastric dilation, perforation, peritonitis, aspiration, and death, emphasizing the potential severity of this condition and the importance of early recognition [2].

In this report, we present a case of Rapunzel syndrome in a 22‐year‐old woman with childhood‐onset trichotillomania and trichophagia requiring surgical intervention. This case highlights the importance of early psychiatric identification and treatment of hairpulling and ingestion behaviors with sustained, targeted behavioral intervention in order to prevent severe medical sequelae.

## 2. Case Presentation

A 22‐year‐old woman with a history of trichotillomania, trichophagia, attention‐deficit/hyperactivity disorder (ADHD), and sensory integration disorder was admitted to the hospital after several weeks of progressive abdominal pain, nausea, early satiety, and bloating. An abdominal CT scan revealed a large intragastric mass consistent with a bezoar (Figure 1).

**Figure 1:**
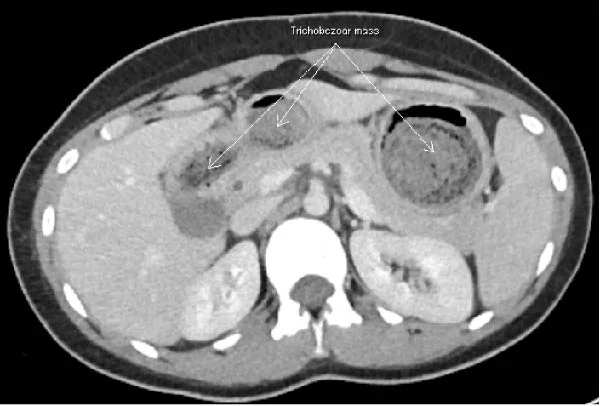
CT abdomen with contrast demonstrating trichobezoar mass extending from gastric body into the duodenum.

Esophagogastroduodenoscopy confirmed a dense, tubular trichobezoar measuring ~2.5 cm in diameter and over 20 cm in length, occupying the gastric body and extending through the pylorus into the third portion of the duodenum, consistent with Rapunzel syndrome (Figure 2). Endoscopic removal attempts were unsuccessful due to the size and density of the mass, necessitating an open gastrotomy with intact removal of the bezoar without surgical complications (Figure 3).

**Figure 2:**
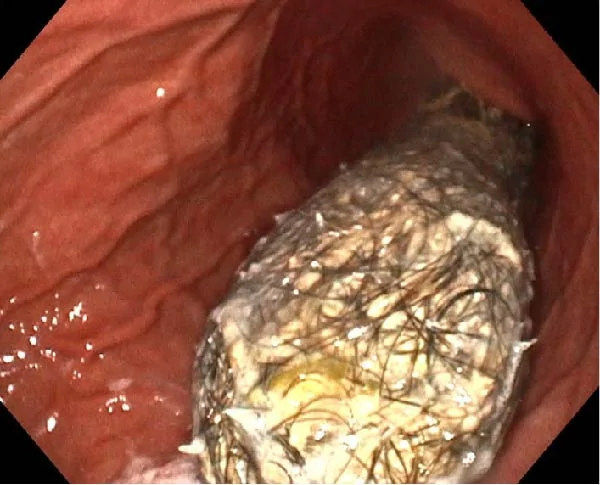
EGD image showing large trichobezoar.

**Figure 3:**
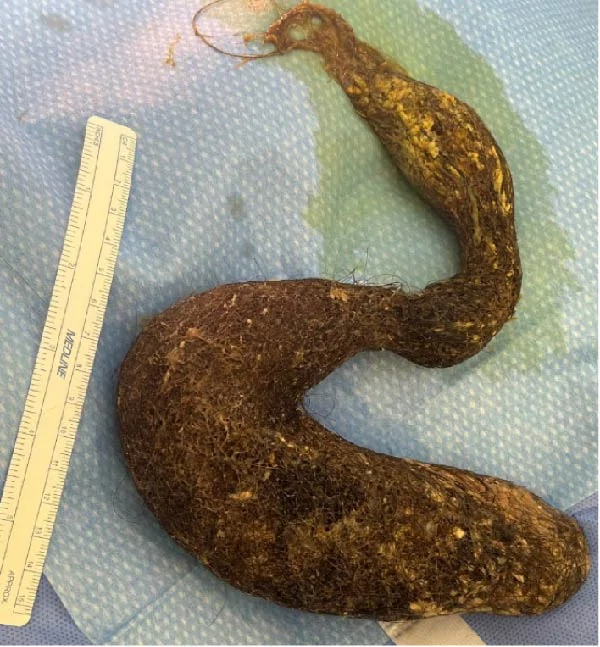
Complete trichobezoar removed after open gastrotomy.

The psychiatry consultation service was engaged on postoperative day one to evaluate her chronic hairpulling and hair‐ingestion behaviors. On examination, she was wearing a wig, and her eyebrows appeared sparse. She was alert, oriented, and cooperative, with normal speech and linear thought processes. Her affect was constricted but appropriate, and her insight into her condition was fair.

The patient reported hairpulling behaviors beginning around ages three to four, initially involving pulling fur from stuffed animals as well as pulling her own hair. Other body‐focused repetitive behaviors, including nail biting and skin picking, were present in childhood but had resolved. The hairpulling behaviors, however, persisted and progressed to insertion of pulled hairs into her nasal cavities, resulting in small intranasal trichobezoars requiring otolaryngologic removal at ages six and seven. Over time, she began habitually ingesting the pulled hairs on a near‐daily basis.

She recalled a period of remission for approximately 2 years during adolescence, during which she abstained in anticipation of school events and photographs. However, the behaviors returned without a clear trigger and intensified over the past several years. She described recurrent urges to pull her hair but denied that there was always discomfort that was followed by a transient sense of relief or satisfaction.

Given the known comorbidity between trichotillomania and obsessive‐compulsive symptoms, the Yale‐Brown Obsessive Compulsive Scale—Second Edition (Y‐BOCS‐II) symptom checklist and severity items were reviewed during the inpatient psychiatric consultation [3]. The patient endorsed tendencies toward perfectionism, cleanliness, and order, as well as obsessional symptoms involving excessive concern or disgust with sticky substances or residues, need for symmetry or exactness, perfectionistic concerns related to appearance or grooming, hoarding or saving concerns, and intrusive meaningless sounds, words, or music. She denied compulsive rituals or avoidance behaviors performed in response to these obsessions and did not describe her hairpulling or hair‐ingestion behaviors as responses to obsessive thoughts. Her Y‐BOCS‐II obsession severity subtotal was 8/25, and her compulsion subtotal was 0/25. Her hairpulling and hair‐ingestion behaviors were therefore considered separately as symptoms of trichotillomania rather than obsessive‐compulsive rituals.

From a developmental standpoint, her history was notable for adoption at 1 year of age, with no available family psychiatric history. Although early developmental milestones were met, her mother described interpersonal difficulties beginning very early in life, including a tendency to become quickly and intensely attached to new adults, as well as engagement in impulsive and risky behaviors persisting into adulthood. Her history was also notable for difficulty completing homework and receipt of a 504 plan throughout schooling, including accommodations for extended time on assignments. Cognitive testing during childhood demonstrated average intellectual functioning, with a WISC‐IV Full Scale IQ score in the 70th percentile. Neuropsychological evaluation in young adulthood supported a diagnosis of ADHD, combined presentation, mild. She completed high school and pursued college‐level education despite ongoing attentional symptoms, which may be explained in part by mild ADHD severity, average cognitive ability, and academic accommodations.

Additional psychiatric history was otherwise unremarkable, with no prior psychiatric hospitalizations, major depressive episodes, or history of self‐injury or suicidal ideation. She had attended outpatient therapy only a few times during childhood. The available history did not identify the specific modality, target symptoms, or clinical outcomes of these visits. There was no evidence of sustained habit reversal training or structured cognitive‐behavioral therapy specifically targeting trichotillomania before hospitalization. Pharmacologic history included a brief trial of sertraline, which was discontinued shortly after initiation due to patient‐reported intolerance, and guanfacine extended‐release for ADHD symptoms, which was discontinued after a short time due to chest pain; no additional pharmacologic ADHD trials were reported. At the time of hospitalization, she reported taking fluoxetine 20 mg daily consistently for more than 8 months, with partial improvement in anxiety but no noticeable improvement in hairpulling or hair‐ingestion behaviors.

Based on the evaluation, the consultation team recommended increasing fluoxetine to 40 mg daily with further titration as tolerated, in addition to initiating outpatient psychotherapy focused on habit reversal training and cognitive‐behavioral therapy targeting trichotillomania.

## 3. Discussion

Rapunzel syndrome represents a rare and serious gastrointestinal complication of trichotillomania with trichophagia and is typically diagnosed only after patients develop significant symptoms. Additionally, high rates of psychiatric comorbidity have been reported in patients with trichotillomania, including obsessive‐compulsive disorder (87%), mood disorders (64%), generalized anxiety disorder (52%), and skin‐picking disorder (44%) [4].

The educational contribution of this case lies in its detailed longitudinal psychiatric characterization of childhood‐onset trichotillomania, with persistent trichophagia progressing silently over many years. The patient’s symptoms began in early childhood and continued in the absence of sustained disorder‐specific behavioral treatment before culminating in Rapunzel syndrome. Clinically, the case highlights the importance of identifying trichophagia as a marker of serious medical risk and considering comorbid ADHD as potentially relevant to the hairpulling and ingestion behaviors themselves through attentional dysregulation, impulsivity, impaired inhibitory control, and automaticity.

A brief comparison with selected prior Rapunzel syndrome reports is provided in Table 1 to highlight the distinctive psychiatric features of the present case.

**Table 1:** Comparison of the present case with selected prior Rapunzel syndrome reports.

Prior report	Patient	Psychiatric/behavioral context	GI presentation/extent	Treatment/follow‐up	How the present case differs
Kouskos et al. [5]	22‐year‐old woman	Trichotillomania and trichophagia	Epigastric pain, nausea, appetite loss; trichobezoar extended from stomach to jejunum	Endoscopic removal failed; open gastrotomy; psychiatry and dietitian referral	Similar age and operative course; present case adds a more detailed longitudinal psychiatric history, including early childhood onset, progression to chronic trichophagia, prior intranasal trichobezoars, and limited sustained disorder‐specific behavioral treatment
Zouaki et al. [6]	16‐year‐old girl	Trichotillomania, trichophagia, and onychophagia were recognized only after further questioning	Abdominal pain, vomiting, tender mass; extension into the third part of the duodenum	Endoscopy unsuccessful; exploratory laparotomy and antral gastrotomy; psychiatry referral	Both cases involved postpyloric extension and failed endoscopic removal; present case adds detailed developmental history, adolescent remission, and Y‐BOCS‐II characterization of obsessional symptoms without compulsions
Forooghi et al. [7]	10‐year‐old girl with ADHD	ADHD and pica‐like hair ingestion	Epigastric pain, nausea, vomiting; substantial gastric trichobezoar	Exploratory laparotomy; psychiatric assessment and behavioral therapy referral	This report is the closest psychiatric comparison because ADHD was also present; the present case extends the discussion into adulthood and provides a more detailed ADHD formulation in relation to impulsivity, impaired inhibitory control, and automaticity
Ahmed et al. [8]	11‐year‐old male child	Trichotillomania, trichophagia, pica; psychosocial context emphasized	Intermittent abdominal pain, hair loss, weight loss; gastric trichobezoar	Surgical removal; psychiatry evaluation; multidisciplinary care advised	The present case similarly emphasizes psychiatric context but adds more detailed differentiation between obsessional symptoms, trichotillomania, trichophagia, and ADHD‐related behavioral features
El‐Mefleh et al. [9]	9‐year‐old girl	Hair‐eating had reportedly ceased before presentation; psychiatric follow‐up was inconsistent	Severe microcytic anemia and epigastric mass; Rapunzel syndrome confirmed on gastroscopy and CT	Laparotomy for bezoar >10 cm; CBT recommended; one series case recurred with poor psychiatric follow‐up	Both reports highlight the risk of long‐standing hairpulling and the importance of psychiatric follow‐up; present case differs by documenting persistent trichophagia into adulthood and emphasizing early recognition before severe medical morbidity develops

Developmentally, this patient’s early adoption history and childhood interpersonal difficulties provide an important context for considering factors that may have contributed to emotional dysregulation and maladaptive coping over time. Prior literature in skin‐picking disorder, a related body‐focused repetitive behavior, has suggested that early maladaptive schemas, including dependence, emotional deprivation, and mistrust, may be associated with symptom severity and with automatic versus focused symptom subtypes [10]. In this case, the patient endorsed recurrent urges to pull her hair but did not consistently describe a classic tension‐relief cycle, suggesting a predominantly automatic pattern of hairpulling. This formulation supports habit reversal training aimed at increasing awareness of early pulling cues and interrupting the behavior before it progresses to ingestion.

Additionally, this patient carried a diagnosis of ADHD, which has been associated with increased prevalence of body‐focused repetitive behaviors. A recent large adult cohort study found that 15.3% of individuals with trichotillomania met criteria for comorbid ADHD and demonstrated significantly higher impulsivity across attentional, motor, and nonplanning domains compared with those without ADHD [11]. Neuroimaging literature in ADHD has implicated fronto–cingulo–striatal networks, including prefrontal and striatal circuits involved in attention regulation and response inhibition [12]. In trichotillomania, diffusion tensor imaging findings have shown reduced white‐matter integrity in anterior cingulate, presupplementary motor, and temporal cortical regions, implicating pathways involved in motor habit generation and suppression [13]. Taken together, these findings suggest that ADHD and trichotillomania may involve overlapping domains of impulsivity, impaired inhibition, automaticity, and habit regulation. In patients with chronic trichotillomania and trichophagia, assessment of ADHD symptoms may therefore help characterize factors contributing to illness persistence, symptom expression, and treatment needs.

The management of trichotillomania remains challenging. Behavioral therapy, particularly habit reversal training, is the recommended gold‐standard treatment. Pharmacologic interventions, including selective serotonin reuptake inhibitors, clomipramine, atypical antipsychotics, and N‐acetylcysteine, have demonstrated inconsistent benefit across studies [14–16]. Emerging neuromodulation approaches, including transcranial magnetic stimulation, have also been explored for trichotillomania. Garip and Yüzgüleç [17] reported improvement in hairpulling symptoms in a three‐patient case series using 20 sessions of transcranial magnetic stimulation, with symptom change measured by the Massachusetts General Hospital Hairpulling Scale. Although preliminary, these findings suggest that transcranial magnetic stimulation may represent a future treatment option for persistent or refractory trichotillomania, though evidence remains insufficient to guide widespread clinical use. In this case, the patient did not receive sustained behavioral treatment in childhood or adolescence, which may have contributed to her illness chronicity and eventual medical complication.

Several limitations should be acknowledged. This report reflects a single case, and causal relationships among the developmental history, psychiatric comorbidity, ADHD, and trichotillomania cannot be established. The psychiatric history was obtained retrospectively during a brief inpatient consultation after surgical management, and the treating team was unable to provide longitudinal outpatient follow‐up. Although the Y‐BOCS‐II symptom checklist and severity items were reviewed, this assessment primarily characterized obsessive‐compulsive symptoms rather than trichotillomania severity. Quantified measures of hairpulling severity, such as the Massachusetts General Hospital Hairpulling Scale, were not administered because of the limited duration and acute medical context of the consultation, limiting objective assessment of baseline symptom severity, treatment response, and recurrence risk. Despite these limitations, this case underscores the importance of early recognition of trichophagia as a high‐risk feature of trichotillomania, along with timely psychiatric assessment, structured symptom monitoring, and referral for behavioral treatment to reduce progressive medical complications.

## 4. Conclusion

Rapunzel syndrome represents an uncommon and severe medical consequence of chronic trichotillomania and trichophagia. In this patient, childhood‐onset trichotillomania progressed silently for years before culminating in a potentially life‐threatening gastrointestinal obstruction. The educational relevance of this case lies in its detailed psychiatric characterization of symptom progression and its emphasis on trichophagia as a high‐risk feature that requires early recognition. For psychiatrists and multidisciplinary clinicians, this case highlights the importance of assessing comorbid psychiatric and neurodevelopmental conditions, including ADHD, when evaluating chronic hairpulling and ingestion behaviors. Early psychiatric identification, structured symptom monitoring, and sustained behavioral treatment remain essential to reducing morbidity, preventing recurrence, and avoiding progressive medical complications.

## Funding

No funding was received for this study.

## Ethics Statement

Institutional review board approval was not required for this single‐patient case report in accordance with the institutional policy. All potentially identifying information has been removed or generalized to protect patient confidentiality.

## Consent

Written informed consent was obtained from the patient for the publication of this case report and accompanying clinical images.

## Conflicts of Interest

The authors declare no conflicts of interest.

## Data Availability

Data sharing is not applicable to this article, as no datasets were generated or analyzed during the current study.
